# Expression of Aberrant MicroRNAs and p16INK4a Associated with HPV (6, 11, 16, 18, 31, 33, 35, 42, 43, 44, 45, 52, 53, and 56) in Oral Dysplasia and Squamous Cell Carcinoma: A Retrospective Study

**DOI:** 10.5146/tjpath.2024.12909

**Published:** 2024-09-02

**Authors:** Layla Hafed, Olfat Shaker, Ghada Ayeldeen, Hatem Amer, Gamilah Al-Qadhi

**Affiliations:** Department of Oral and Maxillofacial Pathology, Faculty of Dentistry, Ahram Canadian University, Giza, Egypt; Department of Oral Pathology, Faculty of Dentistry, Saba University, Sana’a, Yemen; Department of Medical Biochemistry and Molecular Biology, Faculty of Medicine, Cairo University, Cairo, Egypt; Department of Oral and Maxillofacial Pathology, Faculty of Dentistry, Cairo University, Cairo, Egypt; Department of Basic Dental Sciences, Faculty of Dentistry, University of Science and Technology, Yemen

**Keywords:** Oral carcinoma, Oral dysplasia, HPV, MicroRNA, p16^INK4a^

## Abstract

***Objective: *** A few studies indicate that human papillomavirus (HPV) induces aberrant expression of microRNAs (miRNAs) and correlate this with p16INK4a in oral dysplasia (OD) and oral squamous cell carcinoma (OSCC). Therefore, this study aimed to evaluate the expression of miRNA-21, miRNA-22, and miRNA-224 by q-PCR and the p16INK4a by immunohistochemical (IHC) as markers for HPV-positive OSCC and OD in comparison to controls as miRNA expression can be altered by the HPV oncogenes and hence can be used as a biomarker for HPV positive cases.

***Material and Methods:*** Fifty-two specimens were collected from archived paraffin blocks for patients aged between 19 and 88 (31 males and 21 females) from various oral sites. They were examined by IHC using p16INK4a, by RT-PCR for the detection of HPV (6, 11, 16, 18, 31, 33, 35, 42, 43, 44, 45, 52, 53, 56), and by q-PCR for the expression of miRNA-21, miRNA-22, and miRNA-224 in positive specimens.

***Results: *** Out of the 15 OD, three were positive by both techniques. Meanwhile, 17 out of all OSCC specimens showed intense nuclear and cytoplasmic staining by p16INK4a, and only 16 were also positive by RT-PCR. However, all control specimens were negative. MiRNA-21, miRNA-22, and miRNA-224 were overexpressed in 3 specimens of OD and 16 of OSCC.

***Conclusion:*** MiRNA-21, miRNA-22, and miRNA-224, besides p16INK4a, could be used as indicators for HPV-associated OD and OSCC as their expression is attributed to the HPV oncoprotein. Further studies using follow-up data should be done to correlate it with miRNA overexpression.

## INTRODUCTION

Squamous cell carcinoma is derived from the epithelial lining of various anatomic locations in the head and neck region and is known collectively as head and neck squamous cell carcinoma (HNSCC). Tobacco and alcohol use increase the oral, oropharyngeal, hypopharyngeal, and laryngeal cancer risk. Oncogenic viruses are linked to tonsil cancers and the previously mentioned cancers. There is an increase in the incidence of human papillomavirus (HPV)-associated oropharyngeal cancer in developed countries, and the yearly incidence could exceed that of cervical cancer in the coming years (1,2).

Recently, HPV-positive oropharyngeal cancer has dominated the (HNSCC) field and has become increasingly important in terms of diagnostic, preventive, and therapeutic approaches (3). A meta-analysis study has found that HPV infections with high-risk genotypes are more likely to be associated with oral dysplasia and squamous cell carcinoma compared with the normal oral mucosa (4). Compared to tumors with negative HPV, HNSCC patients’ tumors with HPV had better prognoses and survival rates, which may be explained by the existence of an intact p53-mediated apoptotic reaction to chemotherapy-induced stress in the HPV-positive tumors (5,6).

The expression of p16INK4a, a cell cycle regulator and one of the most promising tumor suppressor genes, can be used as a diagnostic and prognostic tool. Specifically, malignant transformation is associated with a lack of p16INK4a expression, which differentiates benign lesions from malignant ones, or it may be associated with p16INK4a overexpression due to the retinoblastoma protein (Rb) pathway alteration so that it can be used as a prognostic tool (7). Studies have shown that p16 hypermethylation is common in oral dysplasia, which promotes oral carcinoma development (8). Light microscopy shows high expression of p16 in some oral dysplastic lesion cells and areas of microinvasion, as well as at the superficial margins of invasive OSCC, which indicates HPV infection (9).

MicroRNAs (miRNAs) are a set of conserved, small, noncoding RNAs known as micro-coordinators of gene expression. They have also become diagnostic and prognostic biomarkers with high specificity and sensitivity (10). The expression of miRNAs is dysregulated in human cancer; alterations in miRNA locations or copy numbers may be the cause. Additionally, dysregulation of transcription factors such as p53 or defects in regulatory proteins can affect miRNA biogenesis (11).

Since many HNSCC patients have little or no history of using tobacco or alcohol, there may be additional risk factors present, such as HPV. It has now become clear that HPV-associated HNSCC is a unique subtype of HNSCC with specific molecular biology. Yang et al. have stated that the HPV genotypes detected by PCR in exfoliated cells and p16 IHC may be more accurate in detecting HPV infection (12).

MiRNA-21, miRNA-22, and miRNA-224 were all dysregulated in many cancers by E6 and E7 HPV oncoproteins (13). Few studies were done on those three miRNAs with OD as well as OSCC (13–19) and correlated with HPV; therefore, the current study was conducted to assess the validity of miRNA-21, miRNA-22, and miRNA-224, as well as p16INK4a expression as biomarkers in HPV-associated OD and OSCC compared to control archived specimens by using real-time PCR and IHC examination. We hypothesize that HPV oncogenes can alter miRNA expression; hence, they can be biomarkers for HPV-positive cases.

## MATERIALS AND METHODS

### Collection and Preparation of Specimens

Fifty-six archival formalin-fixed paraffin-embedded (FFPE) blocks were collected between January 2007 and December 2017. Four specimens from the 56 were excluded due to material loss at the final step (qPCR). Therefore, only 52 specimens were included in this study, and they were grouped as follows: control specimens taken from normal gingiva following gingiva removal for esthetic reasons (n=7), OD specimens (n=15), and specimens of OSCC (n=30). Clinical information was obtained from archived clinical records. Demographic data is shown in Table 1.

**Table 1:** An overview of baseline demographic data.

			**Groups**			**Total**
			**Control group**	**Oral dysplasia (OD) group**	**Oral squamous cell carcinoma (OSCC) group**	
Age (Mean±SD)			44.43±13.03	43.8±11.51	53.6±9.77	
Gender	Female		3 (14.3)	5 (23.8)	13 (61.9)	21 (100)
	Male		4 (12.9)	10 (32.3)	17 (54.8)	31 (100)
Site	Gingiva		7 (63.6)	1 (9.1)	3 (27.3)	11 (100)
	Hard palate		0 (0)	4 (57.1)	3 (42.9)	7 (100)
	Tongue		0 (0)	5 (29.4)	12 (70.6)	17 (100)
	Cheek		0 (0)	2 (22.2)	7 (77.8)	9 (100)
	Floor of the mouth		0 (0)	1 (100)	0 (0)	1 (100)
	Lip		0 (0)	2 (33.3)	4 (66.7)	6 (100)
	Soft palate		0 (0)	0 (0)	1 (100)	1 (100)
Histological grading	Control		7 (100)	0 (0)	0 (0)	7 (100)
	Oral Dysplasia	Mild	0 (0)	8 (100)	0 (0)	8 (100)
		Moderate	0 (0)	3 (100)	0 (0)	3 (100)
		Severe	0 (0)	4 (100)	0 (0)	4 (100)
	Oral squamous cell carcinoma	Well	0 (0)	0 (0)	6 (100)	6 (100)
		Moderate	0 (0)	0 (0)	13 (100)	13 (100)
		Poor	0 (0)	0 (0)	11 (100)	11 (100)

Data were shown as n (%).

From each block, three sections were cut (5 μm thickness). One was mounted on a glass slide and stained by hematoxylin and eosin (H&E) stain, and then each slide was examined by a light microscope to confirm the diagnosis. Grading was done according to the WHO 2022 grading system (20). OD is divided into three grades based on the number of thirds affected by architectural and cytological atypia. Mild dysplasia characterized by atypia limited to the basal third, moderate dysplasia by extension to the middle third, and severe dysplasia by extending to the upper third. However, OSCC were graded based on the degree of differentiation as based on the amount of epithelial nests, cords and islands, keratin pearls, and dysplastic features. Poorly differentiated OSCC demonstrate marked nuclear and cellular pleomorphism, nuclear hyperchromasia, mitotic figures and small islands or individual cells. However, well differentiated OSCC contains large nests of epithelial cells with less atypia (20). Specimen grading was summarized in Table 1. The other two sections were mounted on positively charged slides to improve tissue section adherence during immunohistochemical staining processes. For each FFPE block, ten sections (5 m thick) were cut and immediately deposited in two 1.5 ml microcentrifuge tubes kept at 4 °C for reverse transcription polymerase chain reaction (RT-PCR) and real-time polymerase chain reaction (qPCR).

### Immunohistochemical Staining

An automated immunostainer (AutostainerLink48, Dako, Denmark) was used for immunohistochemical staining. Each section was stained using the ready-to-use, monoclonal mouse antihuman p16INK4a antibody (Clone DO‐7, Code IR616, Dako, Denmark). Each stained section was examined using a low and high-power light microscope (Leica, Switzerland). An image analyzer computer system applying the software Leica Quin 500 (Leica Microsystem, Switzerland) was used to score the positive p16INK4a immunoreaction. To prevent edge artifact, the most homogeneous sections of the reaction were chosen for examination by light microscopy and then transferred to the monitor’s screen. Three independent observers performed evaluations of the immunostained slides using the block-type immunopositivity method for scoring the p16INK4a staining patterns. Sections were categorized as block positive p16INK4a if they had more than 70% strong nuclear with or without cytoplasmic staining (21).

### Preparation of Specimens for RNA Extraction

Firstly, deparaffinization of specimens was done and incubated in 240 μl PKD buffer (commercial product used as digestion buffer) (Qiagen, USA), and then ten μl proteinase was added to it. The prior combination was heated on a heating block for 15 minutes at 56°C, then for 15 minutes at 80°C. After that, the tubes were incubated on ice for 3 minutes and then centrifuged at 12,500 xg for 15 minutes. The supernatants were transferred to a new microcentrifuge tube without distorting the pellet. By using the RNeasy FFPE kit (Qiagen, USA), RNA was extracted following the manufacturer’s instructions and finally treated with DNase I solution (commercial enzyme solutions for DNA digestion) (Qiagen, USA).

### cDNA Synthesis and RT-PCR

According to the manufacturer’s instructions, the quant-script reverse transcriptase was added to the quantiscript RT buffer to the RT primer mix (Qiagen, USA) to prepare the reverse-transcription master mix. After that, each tube containing reverse-transcription master mix received template RNA (14μl), was mixed, and then put down on ice. Then, the mixture was incubated at 42°C in the thermal cycler (Biometra, USA) for 15 minutes. This step was followed by incubating the mixture in a thermal cycler at 95°C for 3 minutes. This was done to inactivate reverse transcriptase. Then 12.5μl of Top Taq was added to 3μl U1 (My09): 5’ CGTCCMARGGAWACTGATC 3’ (negative strand primer) and U2 (My11): 5’ GCMCAGGGWCATAAYAATGG 3’ (positive strand primer where, M=A or C, R=A or G, W=A or T and Y=C or T). After that the mixture was added to 1.5μl cDNA and in a total volume of 2.5μl the mixture was vortexed. The PCR cycling condition was 94°C for 3 minutes, followed by 40 cycles of denaturation at 94°C for 1 minute, annealing at 55°C for 1 minute, and extension at 72°C for 2 minutes. This was followed by an incubation at 72°C for 10 minutes. A broad spectrum primer (universal primer) was used which can detect one or more of the following 14 HPV subtypes (6, 11, 16, 18, 31, 33, 35, 42, 43, 44, 45, 52, 53, and 56) without specification of which one is the positive one. The specimens underwent the prior technique as regards to the marker (50bp).

### qPCR

This procedure was performed only on the positive specimens of IHC and RT-PCR (3 specimens of the OD group, 16 OSCC specimens, and the control specimens) according to the manufacturer’s instructions. RNA was extracted from specimens using Qiagen (Valencia, CA, USA). RNA samples were subjected to RNA quantitation and purity assessment using the NanoDrop® (ND)-1000 spectrophotometer (NanoDrop Technologies, Inc., Wilmington, DE, USA). Reverse transcription was performed on extracted RNA in a final volume of 20-μL RT reactions using the miScript II RT kit (Qiagen, Valencia, CA, USA). Melting curve measurements were carried out following the PCR cycles to confirm the precise production of the anticipated PCR result. MiRNA-21, miRNA-22, and miRNA-224 expression levels were evaluated using the ΔCT method (ΔCT = CT a target gene−CT a reference gene). This method is a way to calculate relative gene expression levels between different samples in that it directly uses the cycles threshold (CTs) generated by the qPCR system for calculation (ΔΔCT = ΔCT (a target sample) −ΔCT (a reference sample)). The final result of this method is presented as the fold change of target gene expression in a target sample relative to a reference sample normalized to a reference gene. The CT value is the number of qPCR cycles needed for the fluorescent signal to cross a specified threshold. Our study calculated ΔCT by subtracting the CT values of control specimens from those of miRNA-21, miRNA-22, and miRNA-224 in OD and OSCC specimens. After that, the ΔCT of the control samples was subtracted from the ΔCT of the OD and OSCC specimens to get the ΔΔCtT. The fold change in miRNA-21, miRNA-22, and miRNA-224 expression was calculated using the 2−ΔΔCT equation.

### Statistical Analysis

Kolmogorov-Smirnov and Shapiro-Wilk tests were used to analyze the data for normality. Data for the p16INK4a, RT-PCR, and q-PCR for miRNA-21, miRNA-22, and miRNA-224 expressions showed non-parametric distribution (non-normal).

Frequencies and percentages followed by chi-square test were calculated for p16INK4a, RT-PCR tests in and between groups.

For each miRNA-21, miRNA-22, and miRNA-224, mean and standard deviation values were calculated in each group dependently. The Independent-Samples Kruskal-Wallis test followed by Mann-Whitney was used to compare two groups in non-related samples. However, the Friedman test was used to repeat the measures, followed by the post hoc Wilcoxon test, which was used to compare more than two groups in related samples. At P ≤ 0.05, the significance level was set. Statistical analysis was carried out using IBM® SPSS® Statistics Version 22.

## RESULTS

### Immunohistochemical Analysis for p16INK4a

**Figure 1:**
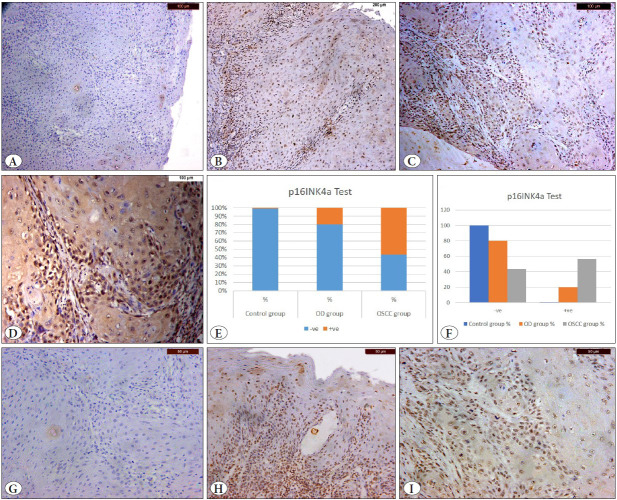


Based on the “block-type” immunopositivity approach for scoring the p16INK4a staining, the immunostaining patterns were block-negative in all 12 OD, 13 OSCC, and control specimens. This was done in consideration that a block-positive p16INK4a immunostaining is one with a strong staining reaction in nuclei with or without cytoplasmic positivity in more than 70% of the tumor cells in the positive specimens (3 OD specimens and 17 OSCC specimens) (Figure 1).

The association between demographic data (gender, site, and histological grading) and p16INK4a results are summarized in Table 2. Regarding gender distribution among different patient groups, there was no significant difference in the negative p16INK4A group (p=0.978). Similarly, there was no significant overall difference in gender distribution for the positive p16INK4A group (p=0.125). Three positive cases of OD specimens were from males. However, 11 cases of the OSCC specimens were from males and six from females.

**Table 2:** Association between p16INK4A expression and demographic data in different groups

**p16INK4A**				**Groups**			**X^2^**	**P-value**
				**Control group**	**OD group**	**OSCC group**		
**Gender in different groups**								
-ve		Female		3 (23.1)	5 (38.5)	5 (38.5)	.045	.978
		Male		4 (21.2)	7 (36.8)	8 (42.1)		
+ve		Female		0 (0)	0 (0)	8 (100)	2.353	.125
		Male		0 (0)	3 (25)	9 (75)		
**Specimens site in different groups**								
-ve	Site	Gingiva		7 (77.8)	1 (11.1)	1 (11.1)	28.655	0.004*
		Hard palate		0 (0)	3 (75)	1 (25)		
		Tongue		0 (0)	3 (33.3)	6 (66.7)		
		Cheek		0 (0)	2 (40)	3 (60)		
		Floor of the mouth		0 (0)	1 (100)	0 (0)		
		Lip		0 (0)	2 (66.7)	1 (33.3)		
		Soft palate		0 (0)	0 (0)	1 (100)		
+ve	Site	Gingiva		0 (0)	0 (0)	2 (100)	3.007	0.557
		Hard palate		0 (0)	1 (33.3)	2 (66.7)		
		Tongue		0 (0)	2 (25)	6 (75)		
		Cheek		0 (0)	0 (0)	4 (100)		
		Lip		0 (0)	0 (0)	3 (100)		
		Soft palate		0 (0)	0 (0)	0 (0)		
**Histological grading in different groups**								
-ve	Grade	Normal		7 (100)	0 (0)	0 (0)	64.00	<0.001*
		OD	Mild	0 (0)	7 (100)	0 (0)		
			Moderate	0 (0)	2 (100)	0 (0)		
			Sever	0 (0)	3 (100)	0 (0)		
		OSCC	Well	0 (0)	0 (0)	2 (100)		
			Moderate	0 (0)	0 (0)	6 (100)		
			Poor	0 (0)	0 (0)	5 (100)		
+ve	Grade	OD	Mild	0 (0)	1 (100)	0 (0)	20.00	0.001*
			Moderate	0 (0)	1 (100)	0 (0)		
			Sever	0 (0)	1 (100)	0 (0)		
		OSCC	Well	0 (0)	0 (0)	4 (100		
			Moderate	0 (0)	0 (0)	7 (100)		
			Poor	0 (0)	0 (0)	6 (100)		

*: significant (p≤ 0.05), **-ve:** negative, **+ve:** positive, **OD:** Oral dysplasia, **OSCC:** Oral squamous cell carcinoma. Data were shown as n (%).

Regarding the site of the lesions, two specimens were from the tongue and one from the hard palate in the OD group. However, the OSCC group showed a higher expression in the tongue (75%) and hard palate (66.7%). There was a significant association between p16INK4A expression and anatomical site in the block-negative group (p<0.005). However, the block-positive group had no significant association with the anatomical site (p>0.05).

The three positive specimens of the OD group were histologically graded as (1 mild dysplasia, one moderate dysplasia, and one severe dysplasia). However, the positive OSCC specimens were differentiated as follows: 6 were poorly differentiated, seven were moderately differentiated, and one was well differentiated. Statistical analysis using Pearson Chi-Square tests revealed a highly significant association between p16INK4A expression and severity levels in the negative group (p<0.001), suggesting varying expression patterns across severity levels. However, in the positive group, there was also significant association with severity levels (p=0.001), indicating differences in expression concerning severity.

A statistically significant difference was found within the OD group in both tests (p=0.02), while there was no statistically significant difference within both control and OSCC groups. There was a significant difference between the groups in negative and positive results, where p= 0.005 and 0.001, respectively. There was also a statistically significant difference between the OD and OSCC groups for the -ve and +ve results where p= 0.027 and 0.011, respectively (Table 3, Figure 1).

**Table 3:** Frequency (N) and percentage (%) for p16^INK4A^ test in different groups.

**Result**	**p16INK4A Test (%**)						
	**Control group**		**OD group**		**OSCC group**		**Total**
	**n**	**%**	**n**	**%**	**n**	**%**	
-Ve	7	100^A^	12	80^Aa^	13	43.3^B^	32
+Ve	0	0^B^	3	20^Bb^	17	56.7^A^	20
Total	7	100	15	100	30	100	52
P-value	-----		0.02*		0.465 ns		
Df	1		1		1		
X2	------		5.400		.0533		

*: Significant (p≤ 0.05), **-ve:** Negative, **+ve:** positive, **df:** Degree of freedom, **N:** Number of cases, **ns:** Non-significant (p>0.05), **OD:** oral dysplasia, **OSCC:** Oral squamous cell carcinoma, **RT- PCR:** Reverse transcription polymerase chain reaction, **X2 :** Chi-square. **Aa and Bb:** Statistically significance difference within the same column. **A and B:** Statistically significance difference within the same row.

### RT-PCR

**Figure 2:**
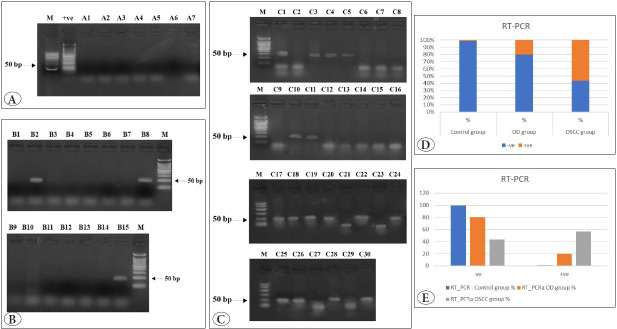


By performing RT-PCR for detection of the HPV gene for the 14 subtypes (6, 11, 16, 18, 31, 33, 35, 42, 43, 44, 45, 52, 53, and 56), bands could not be detected at the same level of the marker 50 bp in all control specimens (lanes A1-A7), which indicated a negative result for the whole subtypes of HPV gene in those specimens (Figure 2). However, bands were noted at the same level of the marker in lanes B3, 8, and 15 of the OD group, indicating HPV gene positivity, which means that one or more of the studied genotypes is present (Figure 2). Bands could also be detected at the same marker level in 16 specimens of the OSCC group, indicating the presence of one or more of the studied HPV genotypes in those specimens (Figure 2). A statistically significant difference was found within the OD group in both tests (p=0.02), while there was no statistically significant difference within the control and OSCC groups. The groups significantly differed in negative and positive results, p= 0.009 and 0.003, respectively. For negative results, there was a statistically significant difference between OD and OSCC groups (p=0.027), while for the positive results, there was a statistically significant difference between OD and OSCC groups (p=0.011) (Table 4, Figure 2).

**Table 4:** Frequency (N) and percentage (%) for RT-PCR test in different groups.

**Result**	**RT-PCR Test (%)**						
	**Control group**		**OD group**		**OSCC group**		**Total**
	**n**	**%**	**n**	**%**	**n**	**%**	
-Ve	7	100^A^	12	80^Aa^	14	46.7^B^	33
+Ve	0	0^B^	3	20^Bb^	16	53.3^A^	19
Total	7	100	15	100	30	100	52
P-value	-----		0.02*		0.715 ns		
Df	1		1		1		
X^2^	------		5.400		0.133		

*: Significant (p≤ 0.05), **-ve:** Negative, **+ve:** positive, **df:** Degree of freedom, **N:** Number of cases, **ns:** Non-significant (p>0.05), **OD:** oral dysplasia, **OSCC:** Oral squamous cell carcinoma, **RT- PCR:** Reverse transcription polymerase chain reaction, **X2 :** Chi-square. **Aa and Bb:** Statistically significance difference within the same column. **A and B:** Statistically significance difference within the same row.

### Q-PCR for miRNA-21, miRNA-22, miRNA-224 Expression

The association between the expression of miRNAs and demographic features (gender, site, and histological grading) was summarized in Table 5. There was no significant association between miRNA expression and gender in the upregulated miRNAs (p=0.149). Also, in the upregulated miRNA cases, there was no significant association between miRNA expression and the site of tissue samples (p=518). However, a significant association was found for the miRNA expression (upregulated levels) among different histological grading in the control, OD, and OSCC groups (p=0.014). In both cases, the p-values were less than 0.05, indicating that the associations are statistically significant and miRNA expression is related to severity.

**Table 5:** Association between different miRNAs expression and demographic data in different groups

**MiRNAs (MiRNA 21, 22, 224) expression**				**Groups**			**X2**	**P-value**
				**Control group**	**OD group**	**OSCC group**		
**Gender in different groups**								
Control			Female	3 (21.4)	5 (35.7)	6 (42.9)	.004	.998
			Male	4 (21.2)	7 (36.8)	8 (42.1)		
Upregulated			Female	0 (0)	0 (0)	7 (100)	2.078	.149
			Male	0 (0)	3 (25)	9 (75)		
**Specimens site in different groups**								
Control	Site		Gingiva	7 (77.8)	1 (11.1)	1 (11.1)	30.038	.003*
			Hard palate	0 (0)	3 (75)	1 (25)		
			Tongue	0 (0)	3 (30)	7 (70)		
			Cheek	0 (0)	2 (40)	3 (60)		
			Floor of the mouth	0 (0)	1 (100)	0 (0)		
			Lip	0 (0)	2 (66.7)	1 (33.3)		
			Soft palate	0 (0)	0 (0)	1 (100)		
Upregulated	Site		Gingiva	0 (0)	0 (0)	2 (100)	3.242	.518
			Hard palate	0 (0)	1 (33.3)	2 (66.7)		
			Tongue	0 (0)	2 (28.6)	5 (71.4)		
			Cheek	0 (0)	0 (0)	4 (100)		
			Lip	0 (0)	0 (0)	3 (100)		
**Histological grading in different groups**								
Control	Grade	Normal		7 (100)	0 (0)	0 (0)	66.00	<.001*
		OD	Mild	0 (0)	7 (100)	0 (0)		
			Moderate	0 (0)	2 (100)	0 (0)		
			Sever	0 (0)	3 (100)	0 (0)		
		OSCC	Well	0 (0)	0 (0)	3 (100)		
			Moderate	0 (0)	0 (0)	6 (100)		
			Poor	0 (0)	0 (0)	5 (100)		
Upregulated	Grade	OD	Mild	0 (0)	1 (100)	0 (0)	19.00	.002*
			Moderate	0 (0)	1 (100)	0 (0)		
			Sever	0 (0)	1 (100)	0 (0)		
		OSCC	Well	0 (0)	0 (0)	3 (100)		
			Moderate	0 (0)	0 (0)	7 (100)		
			Poor	0 (0)	0 (0)	6 (100)		

*: significant (p≤ 0.05), **-ve:** negative, **+ve:** positive, **OD:** Oral dysplasia, **OSCC:** Oral squamous cell carcinoma. Data were shown as n (%).

Quantitative analysis of miRNA levels demonstrated a statistically significant increase in miRNA 21 levels with a 2.6-fold increase in OD specimens and a 21.44-fold increase in OSCC specimens. Furthermore, miRNA 224 levels also showed a statistically significant elevation with a 2.6-fold increase in OD specimens and an 8.3-fold increase in OSCC specimens. On the other hand, the fold change of miRNA 22 was upregulated: 2.4 in the OD specimens and 5.92 in OSCC. However, this fold change was statistically insignificant. Within the same group, there was a statistically significant difference found within the OD group regarding the upregulated miRNA p<0.001, as there was a significant difference between miRNA-21 and miRNA-22 (p=0.5). At the same time, no statistically significant difference was found within both control and OSCC groups.

**Figure 3:**
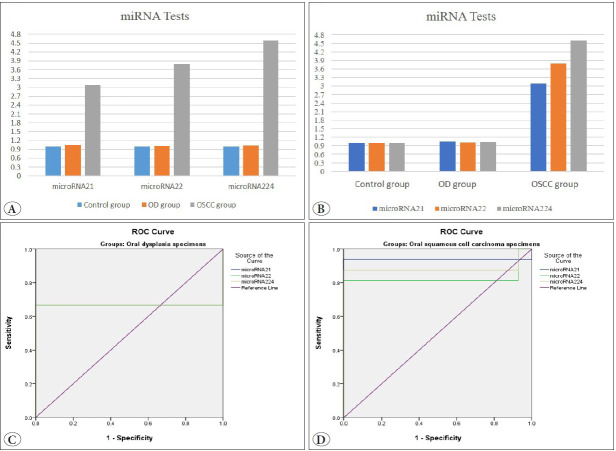


Intergroup comparison showed that miRNA-21 had a significantly upregulated expression between different groups (p=0.019) as there was a significant difference between OSCC and OD groups (p=0.021), while there was no statistically significant difference found within both control and OSCC groups (p=0.67). However, no significant difference was observed between the groups (p=0.14) in miRNA-22 expression. As miRNA-224 was upregulated, a statistically significant difference was found between different groups (p=0.05), and there was a significant difference between the OSCC and OD groups (p=0.042). At the same time, no statistically significant difference was found within either the control and OSCC groups (Table 6, Figure 3).

**Table 6:** The mean, standard deviation values of flexure strength (MPa).

**Variables**	**miRNA-21**		**miRNA-22**		**miRNA-224**		**P-value**
	**Mean**	**SD**	**Mean**	**SD**	**Mean**	**SD**	
Control	1.00^B^	0.00	1.00	0.00	1.00^B^	0.00	---------
OD	1.05^Ab^	0.18	1.02^B^	0.08	1.03^Ab^	0.1	<0.001*
OSCC	3.98A	5.6	3.79	11.04	4.6A	9.72	0.75 ns
P-value	0.019*		0.14 ns		0.05*		

*: Signifiant (p≤ 0.05), **ns:** Non-signifiant (p>0.05), **OD:** Oral dysplasie, **OSCC:** Oral squamous cell carcinoma, **SD:** Standard deviation. **Ab:** Statistically significant difference within the same column. **A and B:** Statistically significant difference within the same row.

According to the ROC curve for OD specimens, it was found that the area under the curve for the three tests used was 0.667, which means that the test sensitivity was fair (66.7%). The cut-off points for miRNA-21, miRNA-22, and miRNA-224 were 1.16, 1.05, and 1.1, respectively. Regarding OSCC specimens, it was found that the areas under the curve for miRNA 21, miRNA 22, and miRNA 224 tests were 0.938, 0.826, and 0.884, respectively. This means the test sensitivity is very good (93%, 82%, and 88%). The cut-off points for these test sensitivities were 1.17, 1.12, and 1.18, respectively (Figure 3).

## DISCUSSION

This study assesses the expression of miRNA-21, miRNA-22, and miRNA-224, as well as p16INK4a indicators for HPV in OD and OSCC compared with control specimens. P16INK4a is frequently used as a reliable bioindicator for high-risk HPV in OSCC and HNSCC, but this is not approved in cases of low-risk HPV infection (22).

In the current study, more than half of the OSCC cases revealed intense nuclear and cytoplasmic staining by p16INK4a and positive expression by RT-PCR. A similar conclusion was reached by Angiero et al. (23). They demonstrated an increase in p16 expression in moderate/severe dysplasia and invasive OSCC, and this positivity increased as the grade of dysplasia progressed. In contrast, a lack of expression appeared in control and mild dysplasia samples (23). Similarly, nuclear and cytoplasmic positive cells were higher in OSCC specimens than in oral leukoplakia (OL) (8). Most HPV-positive OD and OSCC cases had p16 overexpression (12), with a significant association of p16INK4 overexpression in cases of OSCC (23,24).

Alteration of p16INK4a was observed in some HPV-positive head and neck cancers. Overexpression of the viral oncogenes E6 and E7 disrupts p53 and the retinoblastoma protein (pRb) major pathways, resulting in cell cycle dysregulation and apoptosis inhibition (25–27). Subsequently, cyclin-dependent kinase (CDK) inhibitors, including p16INK4a and p21(CIP1/WAF1), are increased, causing a reduction in cyclin D1 and its complex formation with CDK4 (26).

Equally important is the fact that there is a correlation between cell-cycle regulatory proteins such as p53 and p16INK4a in OD and OSCC and telomerase activity. Their findings showed that increased telomerase activity in all PMD and OSCC lesions might be attributable to the clonal expansion of dysplastic cells that harbor p53 and p16INK4a abnormalities (28).

Both IHC and RT-PCR showed positivity in only three OD cases. These findings are in line with a systematic review and meta-analysis that showed significant variability in HPV positivity in OD among the included studies (29). This variability may be due to frequent inactivation of the p16INK4a gene during early carcinogenesis. In non-dysplastic mucosa and oral tumors, however, p16INK4a immunohistochemical expression is frequently absent (30). In non-dysplastic oral epithelium, p16INK4a protein levels are undetectable, as is the case in epithelial dysplasia due to gene inactivation, leading to the absence of positive staining, which is what we found in our control specimens (31).

The average age of HPV-positive patients included in our study was 53.6 years in both the OD and OSCC groups, which is in agreement with Simonato et al. (32), who reported that the positive HPV-associated OSCC cases in their study were older than 60 years and this age is not the most likely age for the HPV positive OSCC which used to be most common in younger patients due to sexual behaviors (32).

In the present study, the number of male-positive cases was higher than that of female-positive cases for HPV. However, there was no statistically significant overall difference in gender distribution (p=0.125), consistent with Emmett et al. (33), who documented a statistically significant increase in HPV-positive oropharyngeal cases among males.

Furthermore, in the present study, most positive cases were from the tongue. This was in agreement with Woods et al. (34), as this site is most commonly affected by HPV. The tongue was also the most commonly associated with miRNA 21 overexpression in a systematic review and meta-analysis conducted by Dioguardi et al. (35). This is in line with our results, as the tongue was the affected site in seven of the cases with upregulated miRNA 21. Contrary to our results, Simonato et al. (32) reported that most of their samples were from the floor of the mouth, which is not the likely anatomical site for the development of HPV-associated OSCC.

Concerning the OD grades, there is a statistical significance among the histological grading of OD and the control group (p <0.001). Similarly, Maheswari et al. (36) reported a statistically significant difference in the upregulation of salivary miRNA-21 in the severe dysplasia group compared to the control group. On the other hand, Di Stasio et al. (37) found that salivary miRNA-21 did not show any variation among OD, control, and OSCC. To our knowledge, there is no documented data on the histological differentiation grading system for OSCC. Emmett et al. (33) reported that positive HPV cases had higher N stage (spread to the lymph node), which explains the significant association of HPV-positive cases with the histological severity levels (p=0.011) in our results.

Regarding miRNAs, numerous studies have shown altered genomic miRNA copy numbers and gene positions to be signs of miRNA dysregulation in cancer cells (amplification, deletion, or translocation) (11). It might also occur due to epigenetic modulation and the dysregulation of a few essential transcription factors, including c-Myc and p53 (11).

MiRNA expression is altered by the HPV-E6 oncogene, especially HPV16, indicating their direct connection (38). Many host dermiRNAs undergo oncogenic HPV regulation in OSCC and OD as miRNA-21, miRNA 22, and miRNA-224 (13).

Current research has shown overexpression of miRNA-21, miRNA-22, and miRNA-224 in OD and OSCC compared to controls. Similarly, miRNA-21 was overexpressed in cancer cases such as colon cancer (39), breast cancer (40), pancreatic cancer (41), and chronic lymphocytic leukemia (42). A study of 540 samples from various cancer patients revealed that miRNA-21 was considerably overexpressed in all of the included solid tumors (43). Specifically, miRNA-21, miRNA-191, and miRNA-17-5p were significantly overexpressed in all tumor types, including breast, colon, lung, pancreas, prostate, and stomach, compared to the normal group (43).

The oncogenic activity of miRNA-21 and its role in regulating various downstream effectors associated with cancer have been discussed in many studies (44). MiRNA-21 knockdown in cultured brain tumor cells activated caspase cascades and increased apoptosis compared to normal tissue, indicating that miRNA-21 functions as an anti-apoptotic agent (45). MiRNA-21 knockdown increases the expression of the phosphatase and tensin homolog (PTEN) tumor suppressor and lowers tumor cell proliferation, migration, and invasion in cultured hepatocellular carcinoma cells. PTEN has been demonstrated to be a direct target of miRNA-21 and contributes to its effects on cell invasion (46).

The miRNA-21 gene is located in the fragile site FRA17B within the 17q23.2 chromosomal region, which is one of the HPV integration loci. The presence of the miRNA-21 gene at or close to HPV integration sites may help to explain why the miRNA-21 gene is upregulated in cervical cancer. HPV integration into the host cell genome leads to genetic and epigenetic alterations (47).

Furthermore, miRNA-21 has been associated with resistance to anti-cancer drugs in head and neck carcinoma cases (15). In the Cisplatin-resistant OSCC group, the expression of STAT3/miRNA-21 was significantly upregulated (p≤0.05) compared to the Cisplatin-sensitive group (15). It was found that the Hyaluronan/CD44 interaction with the novel Nanog and Stat-3 signaling pathway plays a crucial role in miRNA-21 production, which leads to a decline in tumor suppressor protein (PDCD4), an increase in inhibitors of the apoptosis family of proteins (IAPs) and chemoresistance in Hyaluronan-treated HNSCC cells (14). It is now well known that high expression of miRNA-21 is associated with HPV-related cancers, especially in the early stage. Likewise, miRNA21 was upregulated in OD and OSCC specimens in our study, making it useful as a diagnostic or prognostic marker for HPV-associated OSCC (19).

Quercetin inhibits carcinogenesis by modulating the miRNA-22/WNT1/-catenin pathway in OSCC. It enhances miRNA-22 expression and suppresses WNT1 and β-catenin signaling pathways in OSCC cells (18).

Lentivirus mediates miRNA-22 suppression by targeting the NLR family pyrin domain-containing three (NLRP3), causing OSCC cell viability, migration, and invasion (16). However, miRNA-22 is dysregulated by either E6 or E7. This demonstrates that miRNA-22 responds to HPV infection, and their altered expression could be attributed to viral oncoprotein E6 or E7. An expression ratio ≥1.5 of miRNA was informative in distinguishing normal cervix from cervical intraepithelial neoplasia and cervical cancers. This coincides with our results (38)

In contrast to our result, Lu et al. (17) showed that the expression of miRNA224 was significantly downregulated in OSCC tissues. Targeting ADAM17 lowers miRNA-224 production in normal tissues by enabling c-jun binding to its promoter (17).

Previous studies have shown that miRNA-224 could be upregulated or downregulated in different cancers, suggesting that miRNA-224 may have different functions in cancer development depending on the cell type involved (48). Also, the upregulation of miRNA-224 is associated with aggressive progression and poor prognosis in human cervical cancer, and for that, it can also be used as a diagnostic and prognostic marker for HPV-associated tumors such as OSCC (49).

## CONCLUSION

Identifying miRNAs and cell cycle proteins linked with various HPV-mediated cancers is critical since they may be useful as tumor indicators. In this study, HPV-associated OD and OSCC had a distinct positivity profile regarding IHC and RT-PCR and upregulation patterns in miRNA expression, especially miRNA-21, miRNA-22, and miRNA-224, compared to controls. The expression of miRNAs, namely, miRNA-21, miRNA-22, and miRNA-224, and cell cycle regulatory protein (p16 INK4a) could be a promising biomarker in HPV-associated diseases, particularly OD and OSCC. Further studies with large sample sizes and different HPV- DNA detection sets are needed to validate outcome indicators. Also, studying the follow-up data and predicting their prognosis will help to understand the use of miRNAs as a prognostic biomarker for OD and OSCC patients.

## Funding

This research did not receive any specific grant from funding agencies in the public, commercial, or not-for-profit sectors.

## Ethics Committee Approval

This research was approved by the research ethics committee of the Faculty of Oral and Dental Medicine, Ahram Canadian University, Giza, Egypt, in accordance with the Declaration of Helsinki. The database used for this study was accessed with permission from hospitals and institutions.

## Conflict of Interest

All the authors declare that they have no competing interests.

## References

[ref-1] Marur Shanthi, Forastiere Arlene A. (2016). Head and Neck Squamous Cell Carcinoma: Update on Epidemiology, Diagnosis, and Treatment. Mayo Clin Proc.

[ref-2] Johnson Daniel E., Burtness Barbara, Leemans C. René, Lui Vivian Wai Yan, Bauman Julie E., Grandis Jennifer R. (2020). Head and neck squamous cell carcinoma. Nat Rev Dis Primers.

[ref-3] Westra William H. (2009). The changing face of head and neck cancer in the 21st century: the impact of HPV on the epidemiology and pathology of oral cancer. Head Neck Pathol.

[ref-4] Warnakulasuriya Saman (2003). Is human papillomavirus a risk factor for oral squamous cell carcinoma: Is oral infection with human papillomavirus (HPV) a risk factor for oral squamous cell carcinoma (OSCC)?. Evid Based Dent.

[ref-5] Fakhry Carole, Westra William H., Li Sigui, Cmelak Anthony, Ridge John A., Pinto Harlan, Forastiere Arlene, Gillison Maura L. (2008). Improved survival of patients with human papillomavirus-positive head and neck squamous cell carcinoma in a prospective clinical trial. J Natl Cancer Inst.

[ref-6] Ferris Robert L., Martinez Ivan, Sirianni Nicky, Wang Jun, López-Albaitero Andrés, Gollin Susanne M., Johnson Jonas T., Khan Saleem (2005). Human papillomavirus-16 associated squamous cell carcinoma of the head and neck (SCCHN): a natural disease model provides insights into viral carcinogenesis. Eur J Cancer.

[ref-7] Romagosa C., Simonetti S., López-Vicente L., Mazo A., Lleonart M. E., Castellvi J., Cajal S. (2011). p16(Ink4a) overexpression in cancer: a tumor suppressor gene associated with senescence and high-grade tumors. Oncogene.

[ref-8] Agarwal Anshita, Kamboj Mala, Shreedhar Balasundari (2019). "Expression of p16 in oral leukoplakia and oral squamous cell carcinoma and correlation of its expression with individual atypical features". J Oral Biol Craniofac Res.

[ref-9] Natarajan Easwar, Omobono John D., Jones Jonathan C., Rheinwald James G. (2005). Co-expression of p16INK4A and laminin 5 by keratinocytes: a wound-healing response coupling hypermotility with growth arrest that goes awry during epithelial neoplastic progression. J Investig Dermatol Symp Proc.

[ref-10] Faruq Omar, Vecchione Andrea (2015). microRNA: Diagnostic Perspective. Front Med (Lausanne).

[ref-11] Peng Yong, Croce Carlo M. (2016). The role of MicroRNAs in human cancer. Signal Transduct Target Ther.

[ref-12] Yang Li-Qun, Xiao Xuan, Li Chen-Xi, Wu Wen-Yan, Shen Xue-Min, Zhou Zeng-Tong, Fan Yuan, Shi Lin-Jun (2019). Human papillomavirus genotypes and p16 expression in oral leukoplakia and squamous cell carcinoma. Int J Clin Exp Pathol.

[ref-13] Wang Xiaohong, Wang Hsu-Kun, Li Yang, Hafner Markus, Banerjee Nilam Sanjib, Tang Shuang, Briskin Daniel, Meyers Craig, Chow Louise T., Xie Xing, Tuschl Thomas, Zheng Zhi-Ming (2014). microRNAs are biomarkers of oncogenic human papillomavirus infections. Proc Natl Acad Sci U S A.

[ref-14] Bourguignon L. Y. W., Earle C., Wong G., Spevak C. C., Krueger K. (2012). Stem cell marker (Nanog) and Stat-3 signaling promote MicroRNA-21 expression and chemoresistance in hyaluronan/CD44-activated head and neck squamous cell carcinoma cells. Oncogene.

[ref-15] Zhou Xuan, Ren Yu, Liu Aiqin, Jin Rui, Jiang Qingping, Huang Yuanyuan, Kong Lingping, Wang Xudong, Zhang Lun (2014). WP1066 sensitizes oral squamous cell carcinoma cells to cisplatin by targeting STAT3/miR-21 axis. Sci Rep.

[ref-16] Feng Xiaodong, Luo Qingqiong, Wang Han, Zhang Han, Chen Fuxiang (2018). MicroRNA-22 suppresses cell proliferation, migration and invasion in oral squamous cell carcinoma by targeting NLRP3. J Cell Physiol.

[ref-17] Lu Yaoyong, Huang Wendong, Chen Haiwen, Wei Huajun, Luo Aihua, Xia Guangsheng, Deng Xubin, Zhang Gong (2019). MicroRNA-224, negatively regulated by c-jun, inhibits growth and epithelial-to-mesenchymal transition phenotype via targeting ADAM17 in oral squamous cell carcinoma. J Cell Mol Med.

[ref-18] Zhang Chunping, Hao Yuli, Sun Yuanyuan, Liu Ping (2019). Quercetin suppresses the tumorigenesis of oral squamous cell carcinoma by regulating microRNA-22/WNT1/β-catenin axis. J Pharmacol Sci.

[ref-19] Koleśnik Marcin, Stępień Ewa, Polz-Dacewicz Małgorzata (2021). The role of microRNA (miRNA) as a biomarker in HPV and EBV-related cancers. J Pre Clin Clin Res..

[ref-20] WHO Classifcation of Tumours Editorial Board (2022). Head and neck tumours.

[ref-21] Darragh Teresa M., Colgan Terence J., Cox J. Thomas, Heller Debra S., Henry Michael R., Luff Ronald D., McCalmont Timothy, Nayar Ritu, Palefsky Joel M., Stoler Mark H., Wilkinson Edward J., Zaino Richard J., Wilbur David C., Members of LAST Project Work Groups (2012). The Lower Anogenital Squamous Terminology Standardization Project for HPV-Associated Lesions: background and consensus recommendations from the College of American Pathologists and the American Society for Colposcopy and Cervical Pathology. Arch Pathol Lab Med.

[ref-22] Mooren Jeroen J., Gültekin Sibel E., Straetmans Jos M. J. A. A., Haesevoets Annick, Peutz-Kootstra Carine J., Huebbers Christian U., Dienes Hans P., Wieland Ulrike, Ramaekers Frans C. S., Kremer Bernd, Speel Ernst-Jan M., Klussmann Jens P. (2014). P16(INK4A) immunostaining is a strong indicator for high-risk-HPV-associated oropharyngeal carcinomas and dysplasias, but is unreliable to predict low-risk-HPV-infection in head and neck papillomas and laryngeal dysplasias. Int J Cancer.

[ref-23] Angiero Francesca, Berenzi Angiola, Benetti Anna, Rossi Elisa, Del Sordo Rachele, Sidoni Angelo, Stefani Michele, Dessy Enrico (2008). Expression of p16, p53 and Ki-67 proteins in the progression of epithelial dysplasia of the oral cavity. Anticancer Res.

[ref-24] Prakash Pradyot, Khandare Muktesh, Kumar Mohan, Khanna Rahul, Singh Gyan Prakash, Nath Gopal, Gulati Anil Kumar (2013). Immunohistochemical Detection of p16(INK4a) in Leukoplakia and Oral Squamous Cell Carcinoma. J Clin Diagn Res.

[ref-25] Agrawal Gaurav Pralhad, Joshi Priya Shirish, Agrawal Anshita (2013). Role of HPV-16 in Pathogenesis of Oral Epithelial Dysplasia and Oral Squamous Cell Carcinoma and Correlation of p16INK4A Expression in HPV-16 Positive Cases: An Immunohistochemical Study. ISRN Pathology.

[ref-26] Wiest Tina, Schwarz Elisabeth, Enders Christel, Flechtenmacher Christa, Bosch Franz X. (2002). Involvement of intact HPV16 E6/E7 gene expression in head and neck cancers with unaltered p53 status and perturbed pRb cell cycle control. Oncogene.

[ref-27] Li Wei, Thompson Carol H., Cossart Yvonne E., O'Brien Christopher J., McNeil Edward B., Scolyer Richard A., Rose Barbara R. (2004). The expression of key cell cycle markers and presence of human papillomavirus in squamous cell carcinoma of the tonsil. Head Neck.

[ref-28] Abrahao Aline Correa, Bonelli Beatriz Venturi, Nunes Fábio Daumas, Dias Eliane Pedra, Cabral Márcia Grillo (2011). Immunohistochemical expression of p53, p16 and hTERT in oral squamous cell carcinoma and potentially malignant disorders. Braz Oral Res.

[ref-29] Syrjänen S., Lodi G., Bültzingslöwen I., Aliko A., Arduino P., Campisi G., Challacombe S., Ficarra G., Flaitz C., Zhou H. M., Maeda H., Miller C., Jontell M. (2011). Human papillomaviruses in oral carcinoma and oral potentially malignant disorders: a systematic review. Oral Dis.

[ref-30] Gologan Olguta, Barnes E. Leon, Hunt Jennifer L. (2005). Potential diagnostic use of p16INK4A, a new marker that correlates with dysplasia in oral squamoproliferative lesions. Am J Surg Pathol.

[ref-31] Bradley Kyle T., Budnick Steven D., Logani Sanjay (2006). Immunohistochemical detection of p16INK4a in dysplastic lesions of the oral cavity. Mod Pathol.

[ref-32] Simonato Luciana E., Tomo Saygo, Garcia José Fernando, Veronese Luiz Alberto, Miyahara Glauco I. (2016). HPV detection in floor of mouth squamous cell carcinoma by PCR amplification. Jornal Brasileiro de Patologia e Medicina Laboratorial.

[ref-33] Emmett S. E., Stark M. S., Pandeya N., Panizza B., Whiteman D. C., Antonsson A. (2021). MicroRNA expression is associated with human papillomavirus status and prognosis in mucosal head and neck squamous cell carcinomas. Oral Oncol.

[ref-34] Woods Robbie, O'Regan Esther M., Kennedy Susan, Martin Cara, O'Leary John J., Timon Conrad (2014). Role of human papillomavirus in oropharyngeal squamous cell carcinoma: A review. World J Clin Cases.

[ref-35] Dioguardi Mario, Spirito Francesca, Sovereto Diego, Alovisi Mario, Troiano Giuseppe, Aiuto Riccardo, Garcovich Daniele, Crincoli Vito, Laino Luigi, Cazzolla Angela Pia, Caloro Giorgia Apollonia, Di Cosola Michele, Lo Muzio Lorenzo (2022). MicroRNA-21 Expression as a Prognostic Biomarker in Oral Cancer: Systematic Review and Meta-Analysis. Int J Environ Res Public Health.

[ref-36] Uma Maheswari Thirupambaram Natarajasundaram, Nivedhitha Malli Sureshbabu, Ramani Prathiba (2020). Expression profile of salivary micro RNA-21 and 31 in oral potentially malignant disorders. Braz Oral Res.

[ref-37] Di Stasio Dario, Romano Antonio, Boschetti Ciro Emiliano, Montella Marco, Mosca Laura, Lucchese Alberta (2022). Salivary miRNAs Expression in Potentially Malignant Disorders of the Oral Mucosa and Oral Squamous Cell Carcinoma: A Pilot Study on miR-21, miR-27b, and miR-181b. Cancers (Basel).

[ref-38] Wald Abigail I., Hoskins Elizabeth E., Wells Susanne I., Ferris Robert L., Khan Saleem A. (2011). Alteration of microRNA profiles in squamous cell carcinoma of the head and neck cell lines by human papillomavirus. Head Neck.

[ref-39] Feng Yin-Hsun, Wu Chao-Liang, Tsao Chao-Jung, Chang Jan-Gowth, Lu Pei-Jung, Yeh Kun-Tu, Uen Yih-Huei, Lee Jeng-Chang, Shiau Ai-Li (2011). Deregulated expression of sprouty2 and microRNA-21 in human colon cancer: Correlation with the clinical stage of the disease. Cancer Biol Ther.

[ref-40] Wang Hui, Tan Zheqiong, Hu Hui, Liu Hongzhou, Wu Tangwei, Zheng Chao, Wang Xiuling, Luo Zhenzhao, Wang Jing, Liu Shuiyi, Lu Zhongxin, Tu Jiancheng (2019). microRNA-21 promotes breast cancer proliferation and metastasis by targeting LZTFL1. BMC Cancer.

[ref-41] Moriyama Taiki, Ohuchida Kenoki, Mizumoto Kazuhiro, Yu Jun, Sato Norihiro, Nabae Toshinaga, Takahata Shunichi, Toma Hiroki, Nagai Eishi, Tanaka Masao (2009). MicroRNA-21 modulates biological functions of pancreatic cancer cells including their proliferation, invasion, and chemoresistance. Mol Cancer Ther.

[ref-42] Fulci Valerio, Chiaretti Sabina, Goldoni Marina, Azzalin Gianluca, Carucci Nicoletta, Tavolaro Simona, Castellano Leandro, Magrelli Armando, Citarella Franca, Messina Monica, Maggio Roberta, Peragine Nadia, Santangelo Simona, Mauro Francesca Romana, Landgraf Pablo, Tuschl Thomas, Weir David B., Chien Minchen, Russo James J., Ju Jingyue, Sheridan Robert, Sander Chris, Zavolan Mihaela, Guarini Anna, Foà Robin, Macino Giuseppe (2007). Quantitative technologies establish a novel microRNA profile of chronic lymphocytic leukemia. Blood.

[ref-43] Volinia Stefano, Calin George A., Liu Chang-Gong, Ambs Stefan, Cimmino Amelia, Petrocca Fabio, Visone Rosa, Iorio Marilena, Roldo Claudia, Ferracin Manuela, Prueitt Robyn L., Yanaihara Nozumu, Lanza Giovanni, Scarpa Aldo, Vecchione Andrea, Negrini Massimo, Harris Curtis C., Croce Carlo M. (2006). A microRNA expression signature of human solid tumors defines cancer gene targets. Proc Natl Acad Sci U S A.

[ref-44] Feng Yin-Hsun, Tsao Chao-Jung (2016). Emerging role of microRNA-21 in cancer. Biomed Rep.

[ref-45] Chan Jennifer A., Krichevsky Anna M., Kosik Kenneth S. (2005). MicroRNA-21 is an antiapoptotic factor in human glioblastoma cells. Cancer Res.

[ref-46] Meng Fanyin, Henson Roger, Wehbe-Janek Hania, Ghoshal Kalpana, Jacob Samson T., Patel Tushar (2007). MicroRNA-21 regulates expression of the PTEN tumor suppressor gene in human hepatocellular cancer. Gastroenterology.

[ref-47] Thorland Erik C., Myers Shannon L., Gostout Bobbie S., Smith David I. (2003). Common fragile sites are preferential targets for HPV16 integrations in cervical tumors. Oncogene.

[ref-48] Rao Qunxian, Shen Qingli, Zhou Hui, Peng Yongpai, Li Jing, Lin Zhongqiu (2012). Aberrant microRNA expression in human cervical carcinomas. Med Oncol.

[ref-49] Shen Shu-na, Wang Ling-feng, Jia Yong-feng, Hao Yu-qing, Zhang Lin, Wang Hui (2013). Upregulation of microRNA-224 is associated with aggressive progression and poor prognosis in human cervical cancer. Diagn Pathol.

